# The utility of the hematoxylin and eosin staining in patients with suspected Hirschsprung disease

**DOI:** 10.1186/s12893-017-0267-1

**Published:** 2017-06-19

**Authors:** Josephine Amanda Setiadi, Andi Dwihantoro, Kristy Iskandar, Didik Setyo Heriyanto, Gunadi

**Affiliations:** 1 Pediatric Surgery Division, Department of Surgery, Faculty of Medicine, Universitas Gadjah Mada/Dr. Sardjito Hospital, Yogyakarta, 55281 Indonesia; 2 Department of Child Health, Universitas Gadjah Mada Hospital, Yogyakarta, 55291 Indonesia; 3 Department of Anatomical Pathology, Faculty of Medicine, Universitas Gadjah Mada/Dr. Sardjito Hospital, Yogyakarta, 55281 Indonesia; 4 Pediatric Surgery Division, Department of Surgery, Faculty of Medicine, Universitas Gadjah Mada/Dr. Sardjito Hospital, Jl. Kesehatan No. 1, Yogyakarta, 55281 Indonesia

**Keywords:** Diagnosis, Hematoxylin and eosin, Hirschsprung disease, Histopathology, Indonesia, S100

## Abstract

**Background:**

While immunohistochemistry (IHC) methods have been widely conducted for the diagnosis of Hirschsprung disease (HSCR) in developed countries, there are very few studies on their use in developing countries where hematoxylin and eosin (HE) staining is a key element of the diagnosis of HSCR. We aimed to determine the accuracy of HE staining in the diagnosis of HSCR using S100 IHC as the reference standard in Indonesia.

**Methods:**

All histopathology performed for the suspicion of HSCR patients from January 2013 to August 2015 in Dr. Sardjito Hospital, Yogyakarta, Indonesia, were retrospectively reviewed.

**Results:**

Our study included 23 HSCR patients: 9 males and 14 females. The HE staining revealed 14 negative (absence of ganglion cells) cases (61%) and 9 positive (presence of ganglion cells) cases (39%). In S100 IHC, out of the 9 positive cases by HE staining, 6 (67%) were confirmed for having ganglion cells; and out of the 14 negative cases by HE staining, 12 (86%) were reported negative and 2 (14%) were positive by S100 IHC staining. The sensitivity, specificity, positive predictive value, negative predictive value, and accuracy rates of the HE staining were 80% (95% CI: 0.51–0.95), 75% (95% CI: 0.36–0.96), 85.7% (95% CI: 0.56–0.98), 66.7% (95% CI: 0.31–0.91), and 78.3% (95% CI: 0.58–0.90), respectively.

**Conclusions:**

Our study showed that HE staining has relatively moderate accuracy for the diagnosis of HSCR. The use of HE staining is still recommended for the diagnosis of HSCR given the limitation of resource allocation for more expensive IHC technologies in developing countries.

## Background

Hirschsprung disease (HSCR, OMIM #142623), a heterogeneous genetic disorder, is characterized by the absence of ganglion cells in the Meissner and Auerbach plexus of the intestinal tract, resulting in functional bowel obstruction in infants and children [1, 2]. The most widely used classifications for HSCR are as follows: (1) short-segment; (2) long-segment; and (3) total colonic aganglionosis (TCA) [1, 2]. The HSCR incidence differs among ethnic groups: there were 15, 21, and 28 cases per 100,000 live births in Caucasians, Africans, and Asians, respectively [1, 2]. Interestingly, the incidence is higher in the Indonesian population [Karina et al., *under review*]. This pattern might be related to the higher frequency of the *RET* rs2435357 variant in Indonesia [3, 4]. Furthermore, a different genetic characteristic was previously revealed between the Indonesian and Caucasian populations with HSCR [5].

The gold standard for the diagnosis of HSCR is the full-thickness rectal biopsy. While immunohistochemistry (IHC) methods have been widely used for the diagnosis of HSCR in developed countries [6], there are very few studies of their use in developing countries where hematoxylin and eosin (HE) staining is the important element of HSCR diagnosis [7].

Many reports for histopathology findings of HSCR have been described worldwide [6–10]. However, there is a great paucity of knowledge concerning histopathology of HSCR in Indonesia. Therefore, in this study, we aimed to determine the accuracy of HE staining in the diagnosis of HSCR using S100 IHC as the reference standard in Indonesia.

## Methods

All histopathology performed for the suspicion of HSCR in patients who underwent full-thickness rectal biopsy from January 2013 to August 2015 in our hospital were retrospectively reviewed [11].

The Ethical Committee of Faculty of Medicine, Universitas Gadjah Mada/Dr. Sardjito Hospital gave approval for this study (KE/FK/233/EC).

### Full-thickness biopsy

The full-thickness biopsy was performed under general anesthesia. The child was held in the lithotomy position. After aseptic procedures, the anal orifice was held open by an assistant holding two Langenbeck’s retractors. A stay suture was placed on the midline in the posterior rectal wall at least 2 cm above the dentate line. Subsequently, a further stay suture 2 cm higher was placed. A full-thickness strip biopsy of 1–2 cm length was taken between the stay sutures using a sharp-pointed scalpel. Hemostasis was achieved by suturing the defect with a running locking suture from above.

### HE staining and S100 IHC

**Fig. 1:**
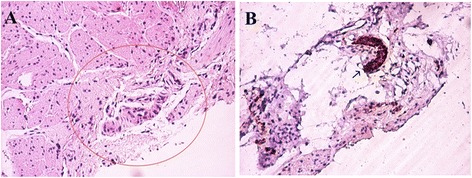
**a** Hematoxylin and eosin staining hypertrophic nerve trunk (Auerbach plexus) in the muscularis layer with no ganglion cells (red circle) in suspected Hirschsprung disease (HSCR). Original magnification × 100. **b** S100 immunohistochemistry showed hypertrophy of nerve trunk and no ganglion cells (arrow) in suspicions of HSCR (× 100)

Histopathological examinations were performed by a senior pathologist at the hospital. The algorithm routinely used in our institute’s pathology laboratory for HSCR diagnosis is HE staining and S100 IHC. We compared the HE staining results in clinically suspicious HSCR patients with the S100 IHC (Fig. 1). The findings of HE staining in HSCR are aganglionosis and frequently connected with hypertrophy of nerve fibers. In non-HSCR tissue, S100 IHC reveals intrinsic nerve fibers and negatively stained ganglion cells surrounded by positive Schwann cells, while in HSCR-affected tissue, intense and prominent S100 IHC shows hypertrophy of nerve fibers [6, 12]. S100 IHC was chosen for comparison since previous studies demonstrated that S100 IHC can effectively and specifically reveal the proliferation of nerve fibers in the HSCR-affected tissue [6, 9, 12, 13]. Furthermore, in the specimen selection process, we excluded the “suspicious” cases (immature, dysplastic ganglion cells) and the insufficient samples. Three to four HE stained levels/sections were examined per specimen.

### Statistical analysis

Data are presented as number and percentages for categorical variables. The HE staining test was evaluated for sensitivity, specificity, positive predictive value, negative predictive value, and accuracy. The McNemar test and Cohen’s Kappa concordance coefficient were used to evaluate the differences in sensitivity and specificity rates between the tests [14, 15]. Sensitivity is the ability of a test to correctly classify an individual as having the disease, while specificity is the ability of a test to correctly classify an individual as not having the disease. Positive predictive value is the proportion of patients with a positive test who have the disease, while negative predictive value is the proportion of patients with a negative test who do not have the disease [16]. Accuracy is the ability of the test to differentiate the patient and healthy cases correctly, while Cohen’s kappa index is a measure of the inter-rater agreement that can account for the probability that raters estimate on at least some variables due to uncertainty [15, 16]. IBM SPSS Statistics version 16 (SPSS Chicago, IL, USA) was used for statistical analysis.

## Results

We studied 23 HSCR patients: 9 males and 14 females. The full-thickness biopsies were performed at the neonatal period, at the age of ≤1 year and at the age of >1 year in eight, nine, and six clinically suspicious HSCR patients, respectively (Table 1).

**Table 1:** Characteristics of Hircshsprung disease patients who underwent full-thickness biopsy

Characteristics	N (%)
Gender	
▪ Male	9 (39)
▪ Female	14 (61)
Age at full-thickness rectal biopsy	
▪ Neonate	8 (35)
▪ ≤1 year old	9 (39)
▪ >1 year old	6 (26)

The HE staining revealed 14 negative (absence of ganglion cells) cases (61%) and 9 positive (presence of ganglion cells) cases (39%). In S100 IHC, out of the 9 positive cases by HE staining, 6 (67%) were confirmed for having ganglion cells; out of the 14 negative cases by HE staining, 12 (86%) were reported negative and 2 (14%) were positive by S100 IHC staining (Table 2).

**Table 2:** Comparison of ganglion cell detection between hematoxylin and eosin (HE) staining and S100 immunohistochemistry in the diagnosis of Hirschsprung disease

HE staining	S100 immunohistochemistry	
	Ganglion cells absent (N, %)	Ganglion cells present (N, %)
Ganglion cells absent (N, %)	12 (52)	2 (9)
Ganglion cells present (N, %)	3 (13)	6 (26)

The sensitivity, specificity, positive predictive value, negative predictive value, and accuracy rates of the HE staining were 80% (95% CI: 0.55–0.93), 75% (95% CI: 0.41–0.93), 85.7% (95% CI: 0.60–0.96), 66.7% (95% CI: 0.35–0.88), and 78.3% (95% CI: 0.58–0.90), respectively (Table 2).

Additionally, we analyzed the sensitivity, specificity, positive predictive value, negative predictive value, and accuracy rates of the HE staining by age group. The sensitivity, specificity, positive predictive value, negative predictive value, and accuracy rates of the HE staining were 100% (95% CI: 0.46–1.00), 66.7% (95% CI: 0.13–0.98), 83.3% (95% CI: 0.44–0.97), 100% (95% CI: 0.34–1.00), and 87.5% (95% CI: 0.65–1.00); 60% (95% CI: 0.17–0.93), 75% (95% CI: 0.22–0.99), 75% (95% CI: 0.30–0.95), 60% (95% CI: 0.23–0.88), and 66.7% (95% CI: 0.36–0.97); and 100% (95% CI: 0.40–1.00), 50% (95% CI: 0.03–0.97), 80% (95% CI: 0.38–0.96), 100% (95% CI: 0.21–1.00), and 83.3% (95% CI: 0.54–1.00) for neonatal, age of ≤1 year and age of >1 year groups, respectively (Table 3).

**Table 3:** Comparison of ganglion cell detection between hematoxylin and eosin (HE) staining and S100 immunohistochemistry in the diagnosis of Hirschsprung disease according to age at full-thickness rectal biopsy

Age at full-thickness rectal biopsy	HE staining	S100 immunohistochemistry	
		Ganglion cells absent (N, %)	Ganglion cells present (N, %)
Neonate	Ganglion cells absent (N, %)	5 (62.5)	1 (12.5)
	Ganglion cells present (N, %)	0	2 (25)
≤1 year old	Ganglion cells absent (N, %)	3 (33)	1 (11)
	Ganglion cells present (N, %)	2 (23)	3 (33)
>1 year old	Ganglion cells absent (N, %)	4 (66)	1 (17)
	Ganglion cells present (N, %)	0	1 (17)

The pairwise comparison of sensitivity and specificity rates did not show a statistically significant difference between HE staining and S100 IHC (*p* = 0.66), whereas the Cohen’s Kappa index was 53.4%. This result indicated that the HE staining and S100 IHC have a moderate agreement to diagnose HSCR. Moreover, the Cohen’s Kappa indexes for different age groups were 71.4% (good agreement), 34.1% (fair agreement), and 57.1% (moderate agreement) for neonatal, age of ≤1 year and age of >1 year groups, respectively.

The ages of the three cases with missed HSCR were 2, 4, and 26-month old, while the ages of the two normal patients misdiagnosed as HSCR were neonate and 6-month old.

The follow-up of those patients who had a positive HE stain is reported as follows: two patients were free of symptoms and did not need further therapy, whereas four patients who were finally diagnosed with hypoganglionosis were managed by pull-through procedure and the pathology results of their surgical specimens were compatible with the initial biopsy results. Among 12 patients with final diagnosis of HSCR, 10 infants underwent a definitive operation while 2 were waiting to pull-through who were on bowel washouts.

## Discussion

Our study found evidence that HE staining has relatively moderate accuracy for the diagnosis of HSCR in Indonesia with the sensitivity and specificity of 80% and 75%, respectively. These findings are compatible with previous studies [17, 18]. Although the diagnostic accuracy of HE staining is relatively lower than that of calretinin and AChE staining, a recent study has revealed that the most frequently used staining methods for rectal biopsies were HE (75.9%) and AChE (73.6%; sensitivity and specificity: 96.8% and 99.4%, respectively), followed by calretinin (33.3%; sensitivity and specificity: 99.1% and 100%, respectively) [7, 10, 19]. Moreover, the use of a variety of complimentary staining tools with HE helps to further increase the diagnostic accuracies by histology, including AChE, calretinin, and S100 [7, 9, 10, 19, 20].

Staining with HE remains the method of choice for the identification of ganglion cells (Fig. 1) [8, 17, 21, 22]. In addition, the advantage of HE staining over IHC is that it only requires fixation of material in buffered formalin and then standard processing, whereas the IHC method requires additional biopsies, freezing of the obtained material as soon as possible, and then processing through various complex procedures [9]. Another disadvantage of IHC is that its implementation in solving problems in anatomical pathology is directly proportional to the experience level of the pathologist who conducts the experiments and the eyes that analyze the findings [23]. Furthermore, the estimated reagents and consumables cost of S100 IHC and AChE staining are $26/sample and $11/sample, respectively, which is more expensive than that for HE staining ($5.5/sample).

Our study revealed that there were no statistically significant differences between HE staining and S100 IHC, however, notably the small sample size of the study implies that a significantly larger sample of patients needs to be involved to better clarify and confirm the results.

Nevertheless, the drawback of HE staining is that most HSCR patients are neonates with only a few small and immature ganglion cells. Consequently, these cells can be easily overlooked in routine frozen sections. In other words, both the IHC and the HE staining have limitations in the diagnosis of immature ganglion cells in newborns [9, 10].

In this study, one of the misdiagnosed HSCR patients was at the neonatal period. Therefore, we suggest that the results of HE staining for suspicious HSCR patients especially at the neonatal period should be interpreted carefully. Furthermore, in countries where other more accurate tests are not available, the patients should be followed up routinely and regularly for the HSCR-related bowel symptoms. Those patients should undergo biopsy again at the age > 28 days to confirm the results of previous biopsy followed by an appropriate management accordingly.

## Conclusions

Our study demonstrated that HE staining has relatively moderate accuracy for the diagnosis of HSCR. The use of HE staining is still recommended for the diagnosis of HSCR given the limitation of resource allocation for more expensive IHC technologies in developing countries.

## Abbreviations

AChE: Acetylcholinesterase
HE: Hematoxylin and eosin
HSCR: Hirschsprung disease
IHC: Immunohistochemistry
